# Research on longitudinal vibration suppression of underwater vehicle shafting based on particle damping

**DOI:** 10.1038/s41598-023-29670-1

**Published:** 2023-02-21

**Authors:** Jie Liu, Tianyang Deng, Xingshan Chang, Feng Sun, Jianhui Zhou

**Affiliations:** 1 School of Naval Architecture, Ocean and Energy Power Engineering, Wuhan University of Technology, Wuhan, 430063 China; 2 Hubei Hangda Science and Technology Co.,Ltd, Wuhan, 430048 China; 3 China Ship Development and Design Center, Wuhan, 430064 China

**Keywords:** Energy science and technology, Materials science

## Abstract

A particle damper is applied to suppress the longitudinal vibration of underwater vehicle shafting in order to reduce vibration level and improve silence and stealth of underwater vehicles. The model of rubber-coated steel particle damper was established with discrete element method and PFC3D simulation software, the damping energy consumption law of collision and friction between particle and damper and between particle and particle investigated, the effects of particle radius, mass filling ratio, cavity length, excitation frequency, excitation amplitude, rotating speed and both stacking and motion states of particles on the system vibration suppression were discussed, and the bench test was carried out to verify the law. It revealed the mechanism of longitudinal vibration suppression of particle damping, established the intrinsic relationship between of total energy consumption of particle and vibration of system, and put forward the evaluating method of longitudinal vibration suppression effect by total energy consumption of particle and vibration reduction ratio. The research results show that the mechanical model of particle damper is reasonable and the simulation data is reliable; the rotating speed, mass filling ratio and cavity length have significant effects on the total energy consumption of particle and vibration reduction ratio.

## Introduction

The underwater vehicle shafting is composed of propeller shaft, stern shaft, intermediate shaft, thrust shaft, bearing, reducer, driving equipment and other devices, which is a continuous elastic body. In operation, the complex axial force of the shafting leads to the axial tension and compression deformation of the shafting, resulting in the common longitudinal vibration of the shafting. The longitudinal vibration of the shafting not only causes the failure of transmission gear and diesel crankshaft and damage of thrust bearing, but also transmits the exciting force to the body of the vehicle. The vibration results in the vibration and radiation of low-frequency noise and greatly affects the sound concealment performance of the underwater vehicle^1^. If the longitudinal vibration of shafting is coupled with other vibrations of shafting, torsional-longitudinal coupled vibration and longitudinal-transverse coupled vibration will be formed, which will seriously affect the safe operation of the shafting.

At present, passive, semi-active and active control methods are used in the longitudinal vibration suppression of underwater vehicle shafting, such as all kinds of shock absorber, damping vibration absorbing devices and so on, but these devices tend to large volume and mass and occupy more space of limited cabins, some still need to provide power energy and complex control. This makes it difficult for the installation, maintenance and reliable operation of the underwater vehicle shafting that requires especially harsh space and load. Therefore, innovative thinking should be carried out to explore new methods, measures and technologies to solve the longitudinal vibration of the shafting, such as the vibration reduction technology of particle damping, so as to improve the mute level and survivability of underwater vehicles.

Particle damping (PD) is a new passive damping technology, which has few changes to the structure of the original system and little increase of additional mass. PD has the advantages of simple structure and wide frequency band of vibration attenuation, and has been widely used in civil engineering, aerospace and mechanical industry. However, its application in longitudinal vibration suppression of ship shafting is rarely reported. In this paper, the rubber-coated steel particle damper is constructed in the middle holes of the shafting to suppress the longitudinal vibration suppression of underwater vehicle shafting. So the vibration energy of the shafting can be lost by the damping generated both collision and friction between particle and cavity and between particle and particle and the material damping of the shafting. At present, the research of particle damping technology mainly includes three aspects: numerical simulation, trial and error test and parameter optimization.

The common simulation methods of vibration suppression based on particle damping include molecular dynamics, particle dynamics, power input and discrete element method. In 1957, the hard ball model and molecular dynamics simulation method was applied to study the fluid state equation, which set a precedent for the study of the macroscopic properties of matter^2^. A particle model was established using molecular dynamics method to simulated the energy consumption characteristics of granular materials and calculate the interaction forces between particles and particles and between particles and boundaries^3^. The particle dynamics method was used to study complex physical properties such as particle damping and energy consumption, establish particle contact model based on Hertz contact theory, and conduct simulation analysis of particle collision process based on finite element^4^. Discrete Element Method (DEM) was proposed for rock mass engineering in 1971, which took into account the interactions between particles and particles and between particles and damper walls, making quantitative analysis of the performance of particle dampers more accurate^5^. Up to now, most DEM are developed based on the disk and spherical particle models, and gradually begin to study the elliptic sphere and massive round particles. Under vertical harmonic excitation, the three-dimensional numerical simulation study on the energy dissipation characteristics of particle dampers was conducted with DEM by controlling the excitation frequency. The study focused on the influence of the acceleration, velocity and displacement amplitudes of vibration on the energy dissipation characteristics of particle dampers^6^. DEM was used to conduct numerical simulation calculation on particle dampers under random excitation, and study the influence of system parameters (such as container size, particle radius and number, mass ratio, external excitation intensity, rebound coefficient and damping ratio of main structure, etc.) on vibration control performance of particle dampers^7^. The energy consumption calculation model of particle collision and friction was established by using DEM to analyze the collision energy consumption mechanism among multiple particles, and determine the solution algorithm of the state, force and energy consumption of particles in the collision process^8^.

In the test study of particle damper, the vibration reduction effect of particle damping in the vertical plane was studied based on the cantilever beam model, which found that particle damping can improve the damping characteristics by nearly 50%^9^. A particle damping vibrator on the wheel tread was installed to attenuate and suppress the irregular vibration of wheel-rail, and the maximum vibration reduction effect was 39 dB^10^. The modal test on a closed cavity arranged particle damping was carried out, the results indicate that the sound pressure of the target field point in the closed cavity drops 3.91dB^11^. A new particle damper of viscoelastic material was proposed and installed at the free end of the cantilever beam to conduct damping effect experiments. The test data show that the damping performance of the damper is better than that of the conventional damper, and it can still work normally under the low frequency vibration ≤ 30 Hz^12^. The simple ball impact damper was applied to improve vibration suppression, and the calculation results of numerical model adopted was consistent with the test results^13^. A damper with soft and hard mixing particles was presented. The test results show that in the low-frequency range, the lower the particle density, the lower the vibration response^14^. The calculation results of 3d DEM was verified by test data, and found that the additional damping of the test device was actually the sum of both impact and friction damping, which resulted in linear attenuation of the amplitude of the primary system^15^. The calculation model of impact damping with particle damping agent was proposed based on the energy dissipation model of elastic–plastic impact of two particles, which was verified by experiments^16^. The application results of additively manufactured structures with different particles in a spindle-tool system in combination was presented. It shows that the structures and particles can reduce the dynamic response and shift the natural frequencies^17^. The experiments of the overall structure with particle damper was applied to verify the feasibility of the damping prediction using the developed coupling procedure^18^.

Under simple harmonic excitation, the effects of particle material, mass ratio and particle radius, etc. on the performance of particle damper was investigated^19^. The rotary elastomer particle damper was prototyped and experimentally tested, and the accuracy of discrete element simulation model is verified, and it was found that the damping torque increases with the increase of packing ratio, rotor speed and elastomer particle size^20^. A cylindrical particle damper was designed to explore the influence of the particle and the different shapes of damping rod head on the buffering energy dissipation characteristics in the impact test^21^. Under vertical harmonic excitation, the influence of excitation amplitude on the energy dissipation characteristics of particle damper was researched^22^. Particle damping was applied to local resonant periodic structure, a composite plate structure with periodic particle damping designed, the vibration transmission characteristics of the structure tested, and the influencing factors and their change rules of the structure explored^23^. Some particles were filled into a rigid shell connected with a vibrator, and the effects of acceleration amplitude, mass ratio, volume and granular material type on the dynamic damping behavior of particles studied experimentally^24^. The research results show that particle mass ratio and material density have an impact on damping performance, and verify the effectiveness of the numerical calculation method^25^. The particle damping simulation technology was introduced into gear driving device, and the simulation results indicate that the particles with a smooth surface have better damping effect at low speed, but the particles with a rough surface had that at high speed. There is no obvious relationship between static friction coefficient and load, which was verified by experiment^26^. Under horizontal and vertical excitation, the characteristics of particle dampers were studied, and carried out numerical simulation and experimental study. The results indicate that the particle damper under vertical excitation has its own characteristics. The shape and size of the damper cavity should be designed according to the acceleration amplitude^27^.

Theoretical analysis, numerical simulation and experiment methods are commonly used to select the parameters of particle damper. Based on the molecular dynamics theory, the quantitative model of energy dissipation of non obstructive particle damping was established, and the calculation results indicate that with the increases of particles diameter, material density, number of granular accumulation layers and vibration strength of the damper, the energy dissipation of particle damping will enhance^28^. The numerical optimization evaluation method for frequent-modulated particle dampers was proposed^29^. The quadratic suitable method was adopted to optimize the mass filling ratio of particle. The study shows that particle damping could significantly reduce the maximum vibration amplitude, and a larger mass filling ratio should be selected^30^. The influence of the main factors on the damper vibration suppression effect was analyzed such as particle diameter, filling rate and both number and installation position of damper, which revealed that under broadband the influence law of various factors on the damper performance of vibration suppression and determined the optimal filling rate (70%) and the optimal installation mode of particle dampers^31^. The influence of major particle parameters (such as granular material, particle radius, filling rate, etc.) on damping ratio was studied, and optimized these characteristic parameters of particle to achieve the best vibration reduction effect^32^. The influence of test parameters on the cutting performance of milling cutters was discussed, an combination scheme of optimal parameter obtained, and verified it^33^. When the packing ratio of steel balls with epoxy granite is 70%, the vibration dissipation time is faster than glass balls, and the damping capacity of the steel balls at optimum packing ratio of 50% is better^34^. The optimization model of particle damping parameter was established, and the optimization of particle damping parameter and practical engineering application of vibration isolation carried out^35^.

At present, some achievements have been made in the simulation calculation, test and optimization of particle damping technology, but most of them are obtained under vertical excitation and no rotating motion, and are often applied in civil engineering, aerospace and mechanical industry, but have not been reported in the shipbuilding industry. In the operation of underwater vehicle shafting, there is not only shafting rotating motion, but also horizontal axial tension and compression motion. In addition, the shafting has strict requirements on the installation space, total mass and quietness in underwater vehicles. Therefore, people pay attention to the research of shafting longitudinal vibration suppression based on particle damping.

The paper is organized as follows: both simulation and test scheme of particle damping, simulation and test results, comparison of simulation and test results, discussion and conclusion.

## Both simulation and test scheme of particle damping

**Figure 1:**
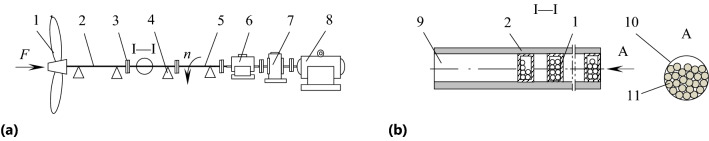
Schematic diagram of underwater vehicle shafting: **(a)** Test device; **(b)** Particle damper. 1—propeller; 2—propeller shaft; 3—connecting flange; 4—bearing; 5—thrust bearing; 6—reducer; 7—drive motor; 8—lightening hole; 9—particle damper; 10—damper cavity; 11—particles.

The underwater vehicle shafting is composed of shafts, bearings and other devices. The external longitudinal excitation force such as the excitation force of propeller and power plant and so on is transmitted to the hull through the shafting and thrust bearing seat, resulting in the longitudinal vibration and radiated noise of the hull. Each shaft section of the shafting is processed with lightening holes, and particle dampers can be installed in them and form a whole with the shaft section. Part of longitudinal vibration energy of the shafting can be consumed with the collision and friction between particles and damper cavity and between particle and particle to suppress longitudinal vibration of the shafting. The schematic diagram of underwater vehicle shafting and particle damper is shown in Fig. 1.

In the research on longitudinal vibration suppression of underwater vehicle shafting, a single particle damper in Fig. 1 was taken as the object to conduct the total energy consumption (TEC) simulation of particle damping and vibration reduction ratio (VRR) test of shafting longitudinal vibration system. In Fig. 1, *F* is the harmonic excitation force, *F* = *A*_0_sin (*ωt*-*φ*); *n* is the rotating speed of shafting.

### Vibration suppression mechanism of particle damping

#### Momentum exchange and consumption of energy by particles

**Figure 2:**
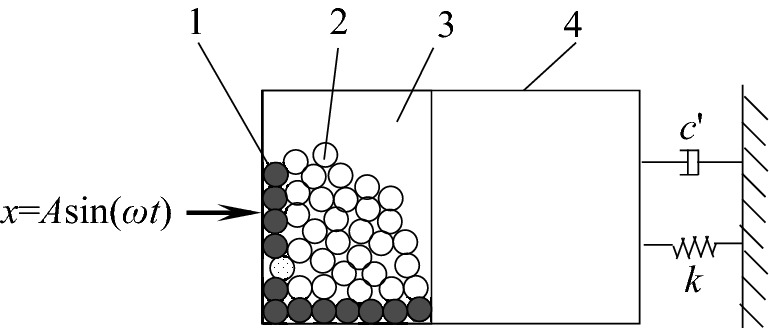
Momentum exchange and consumption of energy. 1—particles of momentum exchange; 2—other particles; 3—particle damper; 4—vibration system.

The TEC consists of the material damping of particle, and the collision damping and friction damping between particles and between particles and damper wall. The vibration suppression of particle damping includes momentum exchange and consumption of vibration energy, which achieved by particles in contact with the vibration system (incluing damper) and paticles as shown in Fig. 2.

In Fig. 2, the particle damper is fixed in the vibration system as a whole; *k*, *c*' and *x* are stiffness, damping coefficient and displacement of the vibration system respectively. When *x* = *A*sin(*ωt*) is applied on a vibration system, momentum exchange occurs between the particle and the vibrating system. It can be seen that (1) some particles (black ones) absorb part of vibration energy of the vibration system by collision and friction between particle and the vibration system; (2) The absorbed vibration energy will be consumed completely or partially by the collision and friction among some particles (black ones) absorbed vibration energy and other particles (white ones). Under ideal conditions, the vibration energy absorbed by particles is completely converted into heat energy and sound energy to release, and the vibration of the system is finally suppressed.

#### Energy consumption calculation of particle damping

In this paper, DEM and PF3D (Particle Flow Code 3D, a three-dimensional computing software developed by Itasca Inc. of America) was used to simulate the characteristics and energy consumption of particle damping.

**Figure 3:**
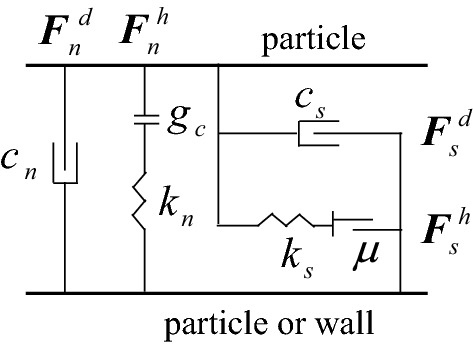
Contact model of particle to particle or wall.

In PFC3D simulation calculation software, the contact state between particles and particles or between particles and damper wall in Fig. 2 is set as Hertz contact, and the contact model is shown in Fig. 3^33^.

In Fig. 3, ${\varvec{F}}_{n}^{d}$ and ${\varvec{F}}_{n}^{h}$ are the normal viscous damping force and Hertz force of the contact surface between particles or between particles and wall respectively;$k_{n}$ and $c_{n}$ are the normal stiffness coefficient and normal damping coefficient of the contact surface respectively; $g_{c}$ is the gap between two particles; ${\varvec{F}}_{s}^{d}$ and ${\varvec{F}}_{s}^{h}$ are tangential viscous damping force and shear force of the contact surface respectively; $k_{s}$ and $c_{s}$ are the tangential stiffness coefficient and tangential damping coefficient of the contact surface respectively; *μ* is the friction factor.

The TEC is obtained by the relation between force and displacement and Newton's second law of motion^36^. Taking any two particles *i* and *j* as examples, their collision energy consumption can be expressed as:

1 $$ \Delta E_{e} = \frac{{m_{i} m_{j} }}{{2(m_{i} + m_{j} )}}(1 - e^{2} )\left| {\Delta v} \right|^{2} $$

where *m* is the mass of particle; *e* is the elastic recovery coefficient of particle, and Δ*v* is the relative velocity of two particles before collision.

Friction energy consumption is determined by the work done by friction force, which can be expressed as:

2 $$ \Delta E_{f} = \mu \left| {F_{n} \delta_{t} } \right| $$

where *µ* is the friction coefficience; *F*_*n*_ is the normal force; *δ*_*t*_ is the tangential relative displacement.

The energy consumption between the particle and the damper wall is also calculated in this way. The TEC of is the sum of the energy consumption of collision and friction between all particles and damper wall, which can be expressed as:

3 $$ E_{{\text{m}}} = \sum {\Delta E_{e} } + \Delta E_{f} $$

#### Parameters of particle damper

The cavity of the particle damper is a cylindrical steel structure, which is composed of a barrel and front and rear walls. The internal diameter of the cavity is *D* = 120 mm, and the distance between the front and rear walls (the length of the cavity) is *L* = 25–75 mm. A certain amount of rubber-coated steel particle are accumulated in the cavity, and the particle core is ordinary steel Q235. In order to reduce the noise radiation generated by collision and friction the particle, the surface of particle is covered with a layer of NBR rubber with thickness 1/6 *R*_*S*_ (spheric radius of particle). Material physical parameters of the cavity and particles of in damper are listed in Table 1.

**Table 1:** Material physical parameters of damper.

Material	Density/(kg/m^3^)	Shear modulus/MPa	Poisson ratio	Normal damping ratio
Barrel and walls	7850	7.923e4	0.30	0.1270
Rubber-coated steel particle	3156	3.03	0.49	0.1653

In the Table 1, the particle density is the equivalent density calculated by the actual mass and volume when the particle radius *R*_S_ = 5 mm.

The interaction parameters between two kinds of materials obtained by experimental test are listed in Table 2.

**Table 2:** Interaction parameters between two kinds of materials.

Material	Recovery coefficient	Static friction coefficient	Normal stiffness/(N/mm)	Damping coefficient	Normal damping ratio
Ordinary steel / Ordinary steel	0.6689	0.20	8706.6	0.3817	0.1270
Rubber-coated steel particle /Rubber-coated steel particle	0.5907	0.50	0.2671	0.4827	0.1653
Rubber / steel	0.6286	0.50	0.2600	0.4336	0.1462

### Parameters of particle damping related to simulation and test

#### Discussion range of relevant parameters

In order to explore the influence of particle damping on vibration energy loss of the system, the discussion range of related parameters is determined as follows: particle radius *R*_S_ = 5, 6, 7, 8, 9 mm; mass filling ratio *δ* = 2.5, 5, 7.5, 10% (The ratio of the total mass of particles to the mass of the damper system); excitation frequency *f* = 3, 4, 5, 10, 20 Hz; excitation displacement amplitude *A* = 1.5, 2, 2.5, 3, 3.5 mm; cavity length *L* = 25, 35, 45, 55, 65, 75 mm; rotating speed *n* = 30, 60, 90, 120, 150 r/min.

#### Parameter value of standard working condition

The parameter value in standard working conditions is *R*_S_ = 5 mm, *δ* = 5%, *f* = 5 Hz, *A* = 3 mm, *L* = 45 mm, *n* = 90 r/min. When discussing the influence of a certain parameter on TEC and VRR, only the value range of the parameter is enlarged, and other parameters remain in standard working conditions.

#### Simulation calculation method of particle damping

The vibration suppression effect of particle damping is closely related to the characteristics and energy consumption mechanism of particle damping. However, the study on the damping mechanism of particle damping becomes more complicated due to the complexity of particle motion and the great uncertainty of particle damping effect caused by different parameter combinations.

**Figure 4:**
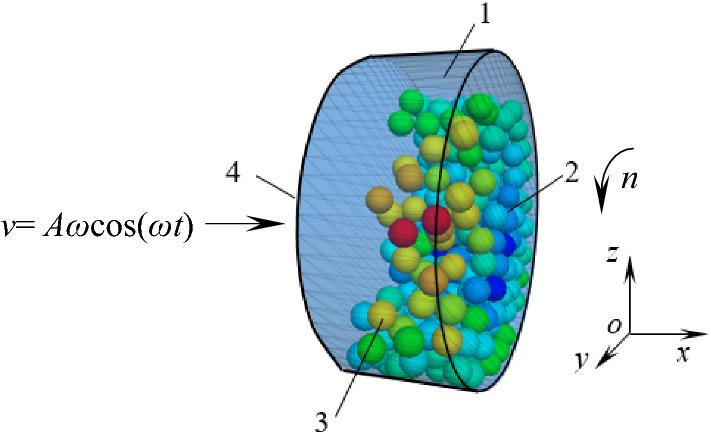
Schematic diagram of particle damper 1—barrel; 2—front wall; 3—particles; 4—rear wall.

Under different working conditions such as cavity rotating and longitudinal vibration, the collision, friction and interaction between particle and cavity and between particle and particle were analyzed, and the effects of particle radius, mass filling ratio, cavity length, excitation frequency, excitation amplitude and rotating speed on TEC were studied. The suppression mechanism of the energy consumption of particle damping (including energy consumption of collision and friction and so on) on longitudinal vibration was revealed. The main process of simulation calculation of particle damping is shown follows:

Define particles. The ball generate command of PFC3D software was used to generate a specified number of particles, and the ball attribute command to assign related attributes, such as particle radius, density, friction coefficient, etc. , and to specify the generation range of particles within the established geometric body through the range command, as shown in Fig. 4.

Geometry and motion characteristics of damper cavity. A damper cavity model is composed of one barrel and both front and rear walls, which is generated with the wall generate command, as shown in Fig. 4. The damper cavity is made of ordinary steel material.

Define the motion characteristics of the cavity. It is assumed that the displacement and motion speed of the cavity in the *x* direction are respectively:

4 $$ \left\{ \begin{gathered} x = A\sin (\omega t) \hfill \\ v = \frac{{{\text{d}}x}}{{{\text{d}}t}} = A\omega \cos (\omega t) \hfill \\ \end{gathered} \right. $$

where *ω* is the excitation frequency; *A* is the excitation displacement amplitude.

The wall. vel. x command in fish language built-in in PFC3D software was used to assign longitudinal velocity *v* to the cavity, and the wall. spin. x command is used to give the rotating speed *n* around *x*-axis in Fig. 4.

Evaluation method of vibration suppression effect. The vibration suppression effect of particle damping depends on the TEC (namely, the sum of energy consumption of particle damping including collision and friction), and the larger the TEC is, the better the vibration suppression effect.

### Test method of vibration suppression of particle damping

The method includes test device of particle damping for vibration suppression, FFT analysis parameter setting and evaluation method of vibration suppression effect.

The test device of particle damping for vibration suppression is self-made by our school, which has rotating motion and longitudinal motion functions. It can simulate the longitudinal vibration suppression test of underwater vehicle shafting, and complete the collecting and processing of relevant test parameters. The test device of particle damping for vibration suppression is mainly composed of variable frequency motor, coupling, rotating speed-torque meter, particle damper, vibration sensor and vibration exciter, etc.

**Figure 5:**
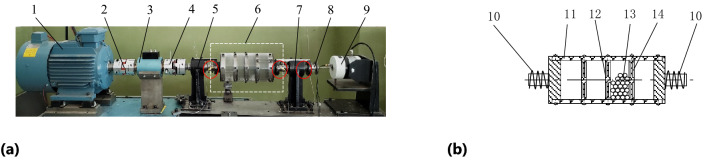
Test device of particle damping for vibration suppression: **(a)** Test device; **(b)** Particle damper. 1—frequency conversion motor; 2, 4—coupling; 3—tachometer; 5, 7—support linear bearing; 6—particle damper; 8—acceleration sensor; 9—vibration exciter; 10—spring; 11—barrel; 12—front wall; 13—particles; 14—rear wall.

The maximum excitation force of high energy shaker HEV-50 is 50 N, frequency range is 0–3 kHz, the maximum amplitude is ± 5 mm, and the force constant is not less than 16 N/A. The power amplifier model is HEAS-50. The vibration test system is composed of Pulse test and analysis software, acceleration sensor and computer developed by B&K in Denmark. Test device of particle damping for vibration suppression is shown in Fig. 5a.

When the test device of particle damping for vibration suppression is running, the vibration exciter 9 outputs current to provide excitation frequency and excitation amplitude (displacement), so as to stimulate the particle damper 6 (Fig. 5b) to produce longitudinal vibration of different frequency and amplitude. Spring 10 (*k*_t_ = 5 N/mm) simulates the system stiffness of the test device and makes the longitudinal displacement recover quickly. The coupling 4 is a rectangular toothed special structure, which can transfer the rotation and axial movement of the damper. The maximum longitudinal moving distance of the coupling 4 can meet the requirements of excitation amplitude. Sensor 8 is the acceleration one. The barrel of particle damper is divided into sections in order to load particles. Front wall 12 and rear wall 14 are fixed on the barrel to form a particle damper, as shown in Fig. 5b.

In the test, parameter settings of FFT analysis at each excitation frequency are listed in Table 3.

**Table 3:** Parameter settings of FFT analysis.

Excitation frequency/Hz	Frequency bandwidth/Hz	Line number	Sampling time/s	Frequency resolution/Hz
3, 4, 5, 6, 7, 8	50	200	4	0.25

It is difficult to evaluate the vibration level of the system based on the variation of vibration energy. Generally, acceleration, velocity and displacement variation are used as evaluation indexes. The vibration suppression effect of particle damping depends on the change state of acceleration speed of the system. The evaluation of longitudinal vibration suppression effect takes the particle-free system (short for cavity system) as the reference object, and the evaluation index is the comparison of the acceleration speed between particle system and cavity system at the dominant frequency (i.e., the maximum amplitude), namely, the VRR is espressed as:

5 $$ \lambda = \frac{{a - a_{{\text{k}}} }}{a} \times 100\% $$

where *a*_k_ and *a* are the acceleration speed of the system with and without particles respectively.

The larger the vibration reduction ratio *λ* is, the more obvious the vibration suppression is.

## Simulation and test results

The influence of particle radius *R*s, mass filling ratio *δ*, cavity length *L*, excitation frequency *ω* and amplitude *A*, and rotating speed *n* on the longitudinal vibration suppression of the system is reflected by the TEC and VRR. The TEC (including the energy consumption of collision and friction) comes from the simulation results, while the VRR comes from the bench test to verify the rationality of the simulation model and the reliability of the simulation data. According to the simulation and test results, the evaluation system of the internal relationship between the TEC and VRR was established.

The influences of the above parameters on the TEC and VRR are analyzed as follows:

### Influence of particle radius on TEC and VRR

**Figure 6:**
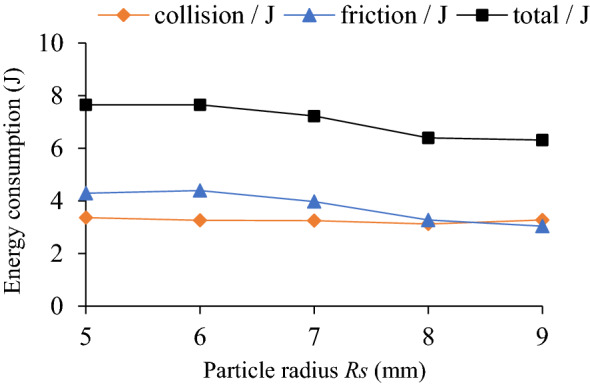
Influence of *R*_S_ on TEC.

**Figure 7:**
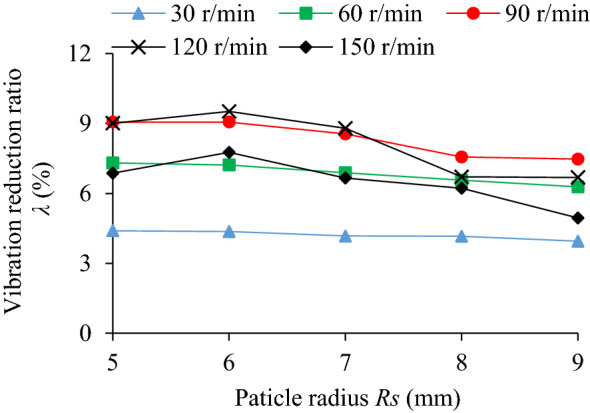
Influence of *R*_S_ on VRR.

**Figure 8:**
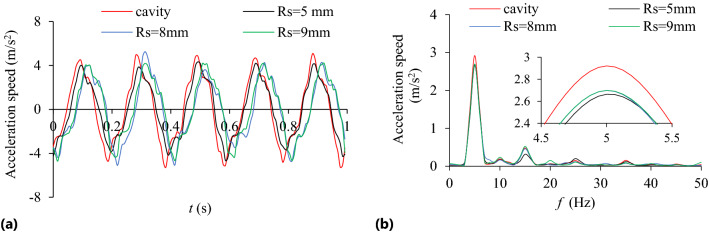
Acceleration speed of system *n* = 90 r/min: **(a)** Time-domain; **(b)** Frequency domain.

The influence of particle radius (*R*_S_ = 5, 6, 7, 8, 9 mm) on the TEC is shown in Fig. 6. The effect of different particle radius and rotating speed on VRR is shown in Fig. 7, and the vibration acceleration of the system at *n* = 90 r/min in Fig. 8.

As can be seen from Figs. 6 and 7, when *R*s = 5 ~ 9 mm and *n* = 90 r/min, the TEC and VRR show obvious vibration suppression effect, and both decrease slightly with the increase of particle radius. At lower rotating speed, such as *n* = 30 r/min, particle radius has little influence on the VRR. Under different rotating speeds the VRR basically reaches the peak value at *R*s = 6 mm, and then decreases with the increase of particle radius. With the increase of the rotating speed, the centrifugal force of the particles in the damper increases, and the number of particles attached to the barrel wall of the damper increase, which leads to the decrease of the number of particles in collision and friction with the particles and the damper, and the reduction of both energy consumption and vibration reduction ratio. For example, when *n* = 150 r/min, the vibration reduction ratio decreases obviously. In Fig. 8, at the dominant frequency of 5 Hz, and the acceleration speed of particle system with different particle radius are lower than that of the cavity system, indicating that the vibration suppression effect is obvious.

Under a certain mass filling ratio, the larger the particle radius is, the larger the mass is, but the less the particle number is. The large mass particles have high friction and kinetic energy, which can increase the energy consumption of friction and collision. However, the reduction of the number of particles may reduce the contact probability between particle and surrounding particles or the cavity, which may affect the energy consumption. And vice versa. Therefore, the interaction of various influencing factors should be considered comprehensively to select the appropriate particle radius. In the case of constant particle radius, the influence of rotating speed on the VRR is complicated and involves the accumulation and motion state of particles, which will be discussed in detail in the following section (Influence of rotating speed on TEC and VRR.).

### Influence of mass filling ratio on TEC and VRR

**Figure 9:**
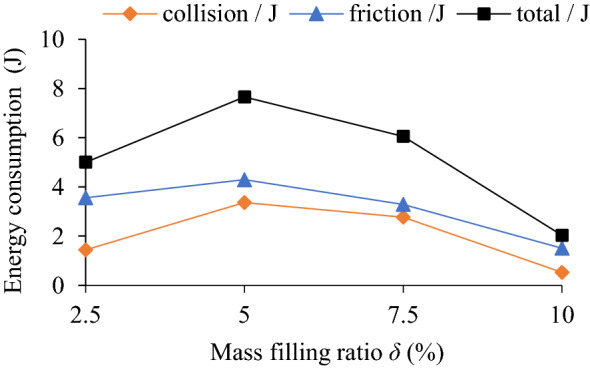
Influence of *δ* on TEC.

**Figure 10:**
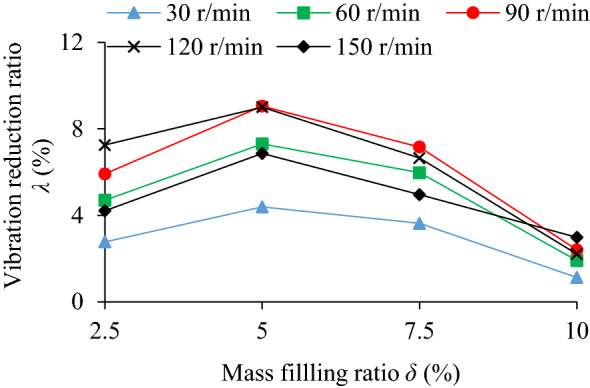
Influence of *δ* on VRR.

**Figure 11:**
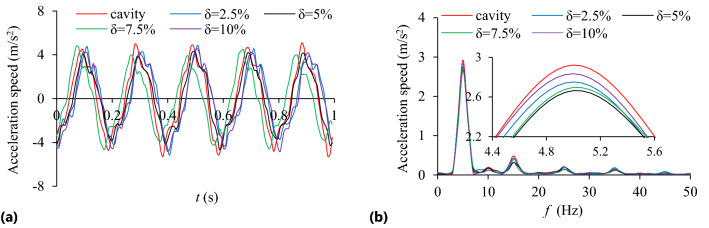
Acceleration speed of system *n* = 90 r/min: **(a)** Time-domain; **(b)** Frequency domain.

The influence of mass filling ratio (*δ* = 2.5%, 5%, 7.5%, 10%) on the TEC is shown in Fig. 9. The influence of different mass filling ratio and rotating speed on the VRR is shown in Fig. 10, and the vibration acceleration speed of the system at *n* = 90 r/min in Fig. 11.

It can be seen from Figs. 9 and 10 that collision energy consumption, friction energy consumption, TEC and VRR have similar variation trends (except *n* = 150 r/min), and they all reach peak values at *δ* = 5% and then show a downward trend. In Fig. 11, when *δ* = 2.5%, 5%, 7.5% and 10%, the VRR of the particle system is lower than that of the cavity system at the dominant frequency of 5 Hz, showing obvious vibration suppression effects.

The mass filling ratio of particles can affects the intensity of collision and friction between particles and cavity. When the mass filling ratio is 2.5%, the total mass and number of particles are relatively little, the probability of collision and friction contact between particle and the wall is reduced so that the TEC is reduced. When mass filling rate rises to 7.5%, the number of particles and the number of accumulation layer are increased. Extruded by the gravity of the upper-layer particles, the clearance among the particles in middle-layer and low-layer is decreased, the movement space of particles is limited, both probability and intensity of collision and friction is declined. When the larger mass filling ratio is, the more obvious the variation trend of energy consumption of the system is, as a result, both collision and friction energy consumption of the system decreases more.

At the same rotating speed, higher or lower mass filling ratio is not conducive to play the role of the energy dissipation of particle damping. Although higher mass filling ratio can increases the total mass and quantity of particles and the static pressure between particles, it leads to the reduction of the movement space of particles, which is not conducive to the collision and friction between particles and wall or between particles. If the mass filling rate is lower, the number of particles is smaller, the momentum exchange between particles and the damper wall is reduced, and the energy consumption of particle damping is also greatly reduced.

### Influence of excitation frequency on TEC and VRR

**Figure 12:**
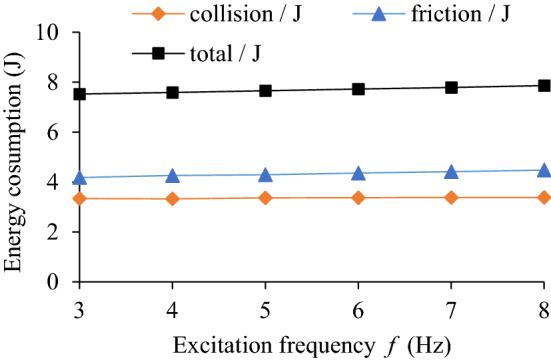
Influence of *f* on TEC.

**Figure 13:**
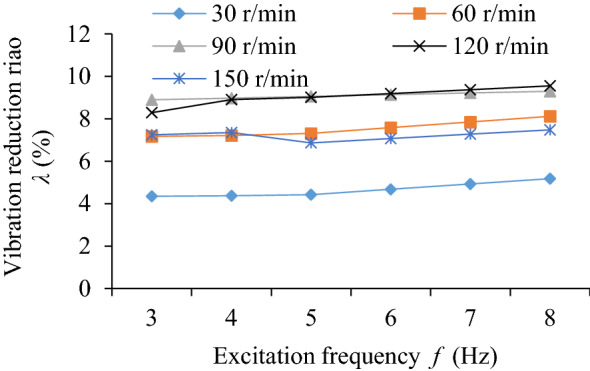
Influence of *f* on VRR.

**Figure 14:**
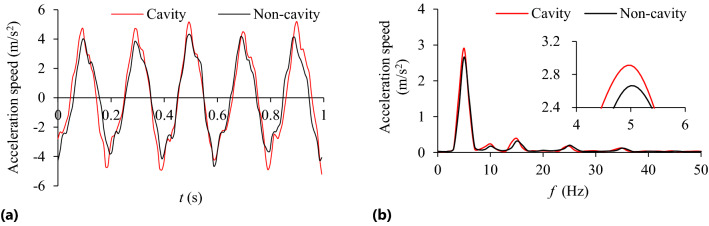
Acceleration speed of system *n* = 90 r/min: **(a)** Time-domain; **(b)** Frequency domain.

The influence of excitation frequency (*f* = 3, 4, 5, 6, 7, 8 Hz) on the TEC is shown in Fig. 12. The influence of different excitation frequencies and rotating speed on VRR is shown in Fig. 13, and the vibration acceleration speed of the system at *n* = 90 r/min in Fig. 14.

From Figs. 12 and 13, it shows that at *n* = 90 r/min, the change trend of the energy consumption of both collision and friction of particles, the variation trend of both total energy consumption of system and vibration reduction ratio of the system are similar, and the trend do not change significantly with the increase of excitation frequency. It shows that the interaction intensity between particles and particles and between particles and cavity basically remains the same, but the friction energy consumption is slightly higher than the collision energy consumption.

It can be seen from Fig. 14 that the acceleration speed of the system with particle (*R*_S_ = 5 mm) near the dominant frequency of 5 Hz is lower than that of the cavity system.

### Influence of excitation amplitude on TEC and VRR

**Figure 15:**
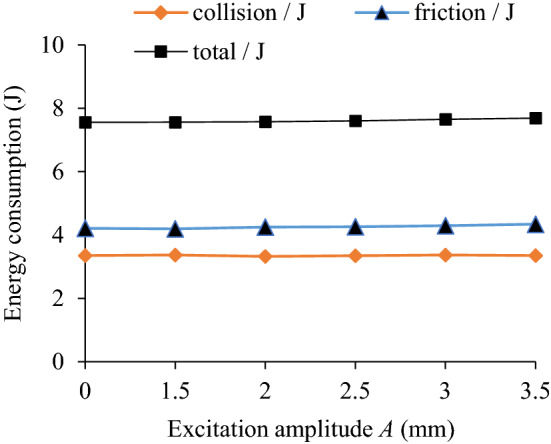
Influence of *A* on TEC.

**Figure 16:**
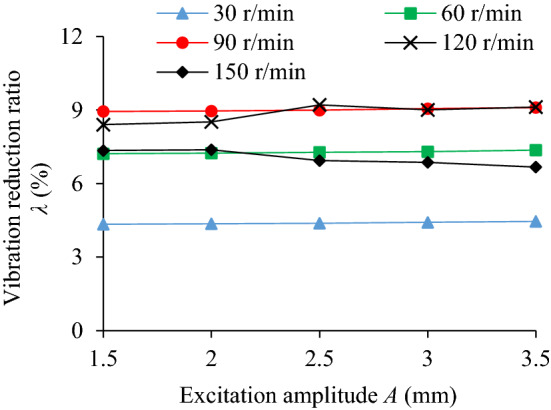
Influence of *A* on VRR.

**Figure 17:**
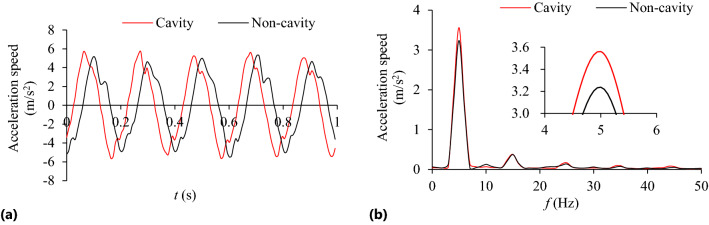
Acceleration speed of system *n* = 90 r/min: **(a)** Time-domain; **(b)** Frequency domain.

The influence of excitation amplitude (*A* = 1.5, 2, 2.5, 3, 3.5 mm) on the TEC is shown in Fig. 15. Under different excitation amplitudes and rotating speeds the VRR is shown in Fig. 16. Taking the excitation amplitude *A* = 3.5 mm as an example, the system acceleration speed at *n* = 90 r/min is shown in Fig. 17.

It can be seen from Figs. 15 and 16 that the excitation amplitude *A* has some influence on the TEC and VRR. At *n* = 90 r/min, with the increase of excitation amplitude, the collision energy consumption, friction energy consumption, the TEC and VRR have a similar variation trend, showing a slight upward trend. When *n* = 150r/min, the reason for the reduction of vibration reduction ratio of the system is the same as the situation in Fig. 7. In Fig. 17, at *A* = 3.5 mm, the acceleration speed of the particle system at the main frequency 5 Hz is lower than that of the cavity system, indicating obvious vibration suppression effect. When the rotating speed *n* is 30, 60, 90 r/min respectively, with the increase of excitation amplitude under the same speed, VRR hardly changes. The results show that though the variation of excitation amplitude can change the velocity and acceleration speed of cavity and particle, and increase the contact probability, collision and friction intensity between particle and the surfaces of barrel and two walls, but the influence degree is limited. Under the same excitation amplitude, the VRR increases with the enhancement of the rotating speed. However, when the rotating speed is increased to 120 r/min and 150 r/min respectively, the variation trend of VRR at two rotating speeds is opposite, and the VRR of the former is higher than that of the latter under the same excitation amplitude. It can be seen that the variation of rotating speed plays a more critical role in VRR, and it can changes the centrifugal force, stacking state and dropping motion state of particles.

### Influence of cavity length on TEC and VRR

**Figure 18:**
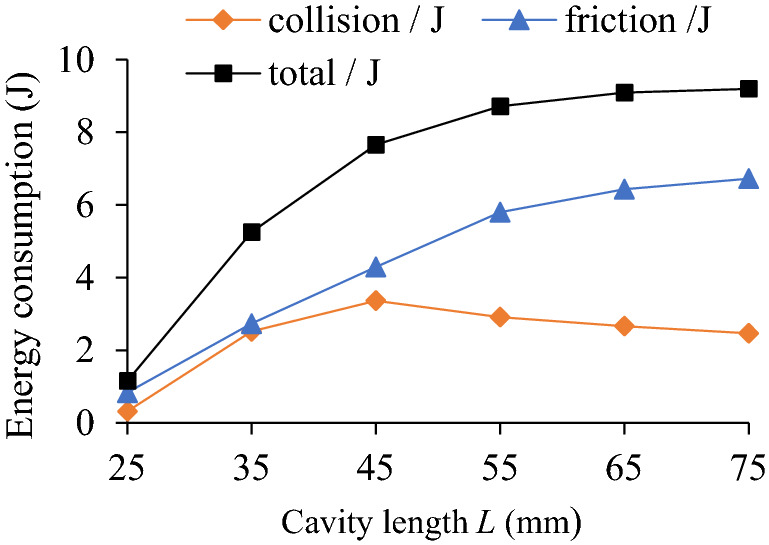
Influence of *L* on TEC.

**Figure 19:**
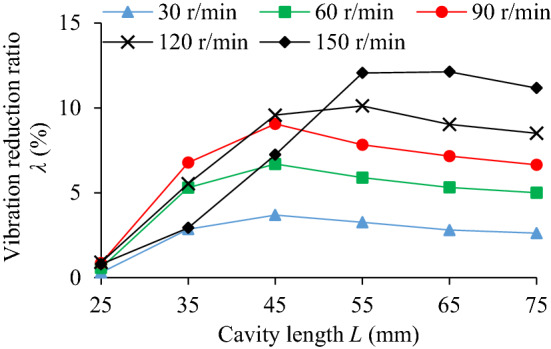
Influence of *L* on VRR.

**Figure 20:**
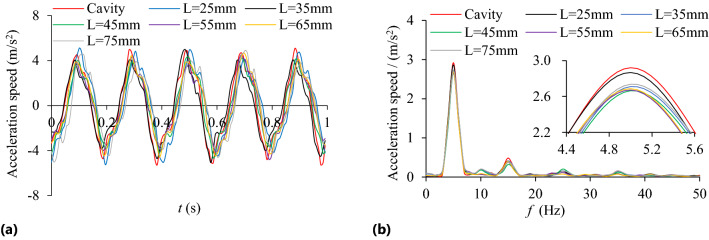
Acceleration speed at *n* = 90 r/min: **(a)** Time-domain; **(b)** Frequency domain.

The influence of cavity length (*L* = 25, 35, 45, 55, 65, 75 mm) on TEC is shown in Fig. 18. Under different cavity lengths and rotating speeds the VRR is shown in Fig. 19, and the system acceleration speed at *n* = 90 r/min in Fig. 20.

As can be seen from Fig. 18, when the cavity length *L* = 25–45 mm, the collision energy consumption increases with the increase of the cavity length and reaches the maximum value at *L* = 45 mm. When the cavity length *L* is greater than 45 mm, the collision energy consumption decreases with the increase of the cavity length. The friction energy consumption increases with the increase of cavity length, but the increasing intensity decreases gradually. The TEC increases with the enhancement of cavity length and tends to be gentle.

In Fig. 19, the TEC increases first and then decreases gradually with the increase of cavity length. When *n* = 90 r/min, the variation trend of the VRR is basically similar to that of the TEC. However, when *L* > 45 mm, the variation trend of the VRR is slightly different, the former decreases slowly, and the latter gradually flattens out. The influence degree is closely related to the change of cavity length.

In Fig. 20, under different cavity lengths (*L* = 25–75 mm), the vibration acceleration speed of the particle system at *n* = 90 r/min is significantly lower than that of the cavity system.

**Figure 21:**
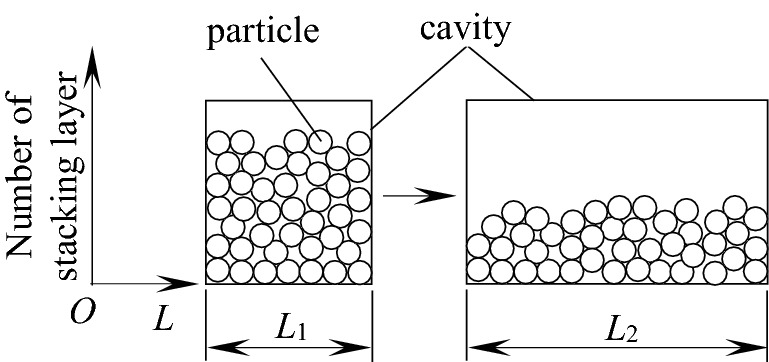
Influence of* L* on stacking state.

Under the condition of a certain mass filling ratio, the variation of cavity length (*L*_1_ ~ *L*_2_) has an important effect on the stacking mode, motion space and motion state of particles (shown as in Fig. 21), thus affecting the TEC and VRR.

It can be seen from Fig. 21 that the cavity length affects the number of stacking layers of particles, the motion state and mechanical properties of upper, middle and lower layers of particles, and ultimately affects the energy consumption level of particles. With the increase of cavity length *L* (*L*_1_ ~ *L*_2_), the number of stacking layers of particles decreases, the distribution of particles is loose, the extrusion pressure among particles decreases, the gap among particles increases, the collision probability between particles and the front and rear walls decreases, the momentum exchange decreases, and the vibration reduction effect of the system is weak. As the clearance among particles increases, the moving space increases, and the friction contact probability between particles and particles and between particles and barrel wall surface also increases correspondingly, so the friction energy consumption of the system has been increasing.

### Influence of rotating speed on TEC and VRR

**Figure 22:**
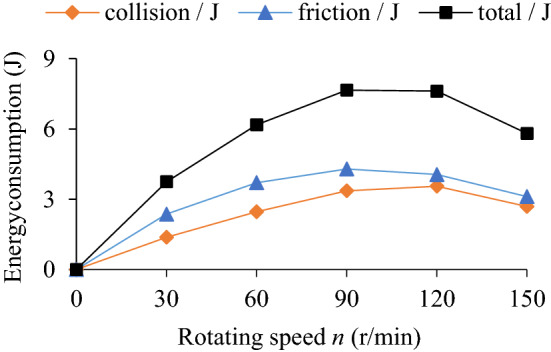
Influence of *n* onTEC.

**Figure 23:**
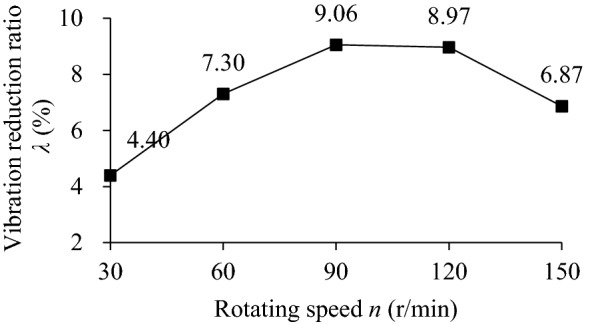
Influence of *n* on VRR.

Figure 22 shows the influence of rotating speed (*n* = 30, 60, 90, 120, 150 r/min) on the TEC. The analysis of the influence of rotating speed on the VRR has been involved in the influence of parameters such as particle radius, mass filling ratio, excitation frequency and excitation amplitude on the VRR. Now, taking the standard working condition (particle radius *R*s = 5 mm in Fig. 7) as an example, Fig. 23 shows the influence of rotating speed (*n* = 30, 60, 90, 120, 150 r/min) on VRR.

As can be seen from Fig. 22, collision energy consumption, friction energy consumption and TEC increase first and then decrease with the increase of rotating speed. At *n* = 0–90 r/min, the energy consumption of collision and friction increases obviously, the former increases slightly more than the latter, and then presents a downward trend. In Fig. 23, the variation trend of VRR with rotating speed is similar to that of the TEC, reaching a maximum of 9.06% at *n* = 90 r/min.

**Figure 24:**
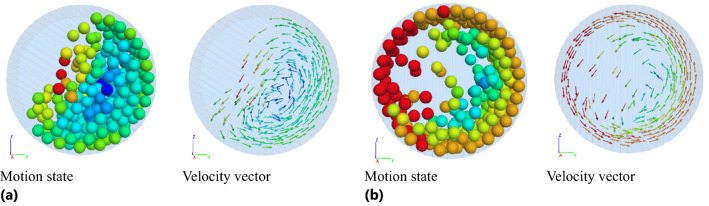
Motion state and velocity vector of particles: **(a)** *n* = 90 r/min **(b)** *n* = 120 r/min.

Under different rotating speed, motion state and velocity vector of particles in damper are shown in Fig. 24.

Increasing the rotating speed (0–90 r/min) can enhance the extrusion, friction and collision between particles and the surface of the barrel, but when the particles lose balance under the action of centrifugal force, gravity, friction and surrounding pressure, the particles will roll down, slide along the damper wall or produce throwing motion. The friction and collision probability between particles and walls, between particles and barrel, and between particles and particles increases, and the TEC and VRR are enhanced. The motion state and velocity vector of particles are shown in Fig. 24a.

However, when the rotating speed exceeds 90 r/min, the increase of centrifugal force of particles leads to some particles sticking close to the wall of the barrel, which reduces the drop motion of particles and the contact probability between particles, and the TEC begins to decline. When rotating speed of more than 120 r/min, particles attached to the wall of the barrel increase due to the further increase of the centrifugal force, at this time only the inner particles do fall movement, which leads to the friction and collision between particle and particle and between particles and wall reduced correspondingly, thus presents the downward trend of energy consumption. The motion state and the velocity vector of particles at *n* = 120 r/min is shown in Fig. 24b.

## Comparison of simulation and test results

Taking rotating speed *n* = 90 r/min for instance, the simulation and test results of particle damping for vibration suppression show that the variation trends of both results are basically similar with particle radius, mass filling ratio, excitation frequency, excitation amplitude, cavity length and rotating speed. The influence of particle radius, mass filling ratio, cavity length and rotating speed on the system vibration suppression effect is greater, and the influence of excitation frequency and excitation amplitude weaker, as shown in Table 4.

**Table 4:** Variation rule of TEC and VRR at *n* = 90 r/min.

Influencing parameter	Variation rule of TEC and VRR	Parameter influence level	Evaluation
Particle radius *R*_S_	It decreases slightly with the increase of particle radius	Greater	The change trend of the simulation and test results basically similar
Mass filling ratio *δ*	With the increase of mass filling ratio, it first increases and then decreases (except *n* = 150 r/min and *R*s = 2.5 mm)	Greater	
Excitation frequency *f*	It increases slightly with the increase of excitation frequency	Weaker	
Excitation amplitude *A*	It basically keeps constant with the increase of excitation amplitude	Weaker	
Cavity length *L*	It first increases and then decreases with the increase of cavity length	Greater	
Rotating speed *n*	With the increase of rotational speed, it first increases and then decreases gradually	Greater	

As can be seen from Table 4, in the influencing parameter such as particle radius *R*_S_, mass filling ratio *δ*, excitation frequency *f*, excitation amplitude *A*, cavity length *L*, rotating speed *n* (except *n* = 150 r/min and *R*s = 2.5), the variation trend of VRR is basically consistent with the TEC, which verifies the rationality of the simulation model and the reliability of simulation results and reveals internal relationship between VRR and TEC.

## Discussion

The vibration suppression effect of particle damping involves the energy produced by vibration and the energy consumed by particle damping.

### Vibration energy of the system

The vibration period of the system is *T*, and the vibration energy *E* of one period (*T* = 2π/ω) is the sum of kinetic energy *E*_d_ and potential energy *E*_s_ of one period.

The expressions for *E*_d_, *E*_s_ and *E* are as follows respectively.

6 $$ \left\{ \begin{gathered} E_{{\text{d}}} = \frac{1}{2}mv^{2} \hfill \\ E_{{\text{s}}} = \frac{1}{2}kx^{2} \hfill \\ E = E_{{\text{d}}} + E_{{\text{s}}} = \int_{0}^{{\frac{2\pi }{\omega }}} {\left( {\frac{1}{2}mv^{2} + \frac{1}{2}kx^{2} } \right)} {\text{d}}t \hfill \\ = \int_{0}^{{\frac{2\pi }{\omega }}} {\left( {\frac{1}{2}mA^{2} \omega^{2} \cos^{2} (\omega t) + \frac{1}{2}kA^{2} \sin^{2} (\omega t)){\text{d}}t} \right)} \hfill \\ \end{gathered} \right. $$

where *m* is the system mass; *v* is the motion speed of the vibration system, as shown in Formula (4), $v = \frac{{{\text{d}}x}}{{{\text{d}}t}} = A\omega \cos (\omega t)$.

It can be seen from Formula (6) that the vibration energy of the system is related to the system mass *m*, equivalent stiffness *k*, excitation frequency *ω* and excitation amplitude *A*. Among them, the system mass* m* and equivalent stiffness *k* affect the natural frequency *ω*_*n*_ of the system, which can be expressed as

7 $$ \omega_{{\text{n}}} = \sqrt{\frac{k}{m}}  $$

It can be obtained from Formula (7)

8 $$ k{ = }\omega_{{\text{n}}}^{2} m $$

When resonance occurs, the vibration energy of the system reaches its maximum. At resonance point, *ω* = *ω*_*n*_, then, the instantaneous vibration energy *E*(*ω*_*n*_) of the system can be expressed as

9 $$ E\ominus \omega_{n} \ominus = \frac{{\uppi kA^{2} }}{{\omega_{n} }} $$

### Damping energy consumption of the system

Taking the vibration system in Fig. 2 as an example, the vibration suppression comes from the energy consumption generated separately by the material damping, collision damping and friction damping of the vibration system. The sum of these energy consumption can be expressed as:

10 $$ E^{\prime} = \Delta E_{1} { + }\Delta E_{2} + \Delta E_{3} $$

where $\Delta E_{1}$, $\Delta E_{2}$ and $\Delta E_{3}$ are respectively the energy consumption of internal damping of materials, collision damping and friction damping of the vibration system.

Supposing the equivalent viscous damping force *F*_c_ of the vibration system is:

11 $$ F_{{\text{c}}} = c^{\prime}v $$

where *c*' is the equivalent damping coefficient of the vibration system, *c*' = *c*_m_ + *c*_n_, therein *c*_m_ and *c*_n_ are equivalent damping coefficients of particle and vibration cavity system (particle-free system) respectively.

In one period (*T* = 2π/ω), the energy *E*' consumed by *F*_c_ can be expressed as:

12 $$ \begin{aligned} E^{\prime} & = \int_{0}^{{\frac{2\pi }{\omega }}} {F_{c} } {\text{d}}x = \int_{0}^{{\frac{2\pi }{\omega }}} {c^{\prime}} v{\text{d}}x = \int_{0}^{{\frac{2\pi }{\omega }}} {c^{\prime}} v\frac{{{\text{d}}x}}{{{\text{d}}t}}{\text{d}}t = \int_{0}^{{\frac{2\pi }{\omega }}} {c^{\prime}v^{2} } {\text{d}}t = c^{\prime}\omega A^{2} \int_{0}^{2\pi } {\cos^{2} (\omega t)} {\text{d}}\omega t \\ & = c^{\prime}\omega A^{2} \int_{0}^{2\pi } {\frac{1}{2}[1 + \cos 2(\omega t)]} {\text{d}}\omega t = \pi c^{\prime}\omega A^{2} \\ \end{aligned} $$

Substitute *c*' = *c*_m_ + *c*_n_ into Formula (12) to get

13 $$ E^{\prime} = \pi c^{\prime}\omega A^{2} = \pi (c_{{\text{m}}} + c_{{\text{n}}} )\omega A^{2} = \pi c_{{\text{m}}} \omega A^{2} + \pi c_{{\text{n}}} \omega A^{2} = E_{{\text{m}}} + E_{{\text{n}}} $$

where *E*_m_ is TEC of particle; *E*_n_ is TEC of the particle-free system.

When TEC of particle *E*_m_ > 0, it shows that particle damping can suppress the vibration of the system, the vibration suppression effect in resonance can be represented by the ratio of the two parameters (*E*_m_ and *E*(*ω*_*n*_) ), namely the loss factor in damping *η*.

14 $$ \eta = \frac{{E_{{\text{m}}} }}{{E(\omega_{n} )}} = \frac{{\pi c_{{\text{m}}} \omega A^{2} }}{{\frac{{\pi kA^{2} }}{{\omega_{n} }}}} = \frac{{c_{{\text{m}}} \omega \omega_{n} }}{k} $$

According to Formula (12), it shows that the damping energy consumption of the vibration system *E*' is related to the equivalent damping coefficient *c*' of the vibration system, excitation frequency *ω* and excitation amplitude *A*. In resonance, the vibration energy of the vibration system *E*(*ω*_*n*_) is related to the equivalent stiffness *k* and excitation amplitude *A* of the vibration system as shown in Formula (9). In Formula (9) ~ (14), it can be seen that there is a close relationship among *E*', *E*_m_ and *E*(*ω*_*n*_), the larger *η* is, the more obvious the vibration suppression effect of particle damping is. The *η* increases as *c*_m_, *ω* and *ω*_*n*_ augment and decreases as *k* increases.

When the excitation frequency is the same as or close to the resonance one, the vibration suppression effect of the damping is more distinct. When particles collide and rub with damper wall, the plastic deformation (collision damping) or friction heating (friction damping) produced consumes part of the vibration energy of the system, limiting the change degree of movement speed of the system. At the same time, some particles also get some energy from the contact with the damper wall, and proceed the momentum exchange and energy consumption with other particles (or damper wall). The ideal situation is that all the energy obtained by the contact between particle and damper wall is consumed by the collision and friction with other particles, resulting in a significant vibration suppression effect. However, the vibration suppression effect of particle damping is affected by many factors: the internal factors mainly include the quantity, mass, structure size of particles and damper cavity, and mechanical properties of materials; the external factors mainly include excitation frequency, excitation amplitude and rotating speed. Under the interaction of many factor-levels, the situation of momentum exchange and energy consumption between particles and damper cavity and between particles is complicated. The larger collision contact force, the longer collision time, the greater friction force and the smaller recovery coefficient can make the dissipation effect of vibration energy of the system more noticeable.

In the simulation calculation and test verification, the damper has both longitudinal simple harmonic vibration and circumferential rotating motion. Therefore, in rotating motion, the particles will also roll or slide on the damper wall, and even throw down to impact the top particles and the damper wall, may sometimes appear that a part of the particles is involved in collision and friction or a part of them is completely at rest. The motion state of particle indicates that particle damping is highly nonlinear and difficult to describe the energy dissipation mechanism of various kinds of damping accurately by theoretical model.

### Characterization of longitudinal vibration suppression effect of particle damping

The longitudinal vibration suppression effect of particle damping can be represented by loss factor in damping *η*. However, the TEC of particle is restricted by many parameters, including equivalent damping coefficient, excitation frequency and excitation amplitude and so on. The vibration energy of the system is relative to the mass, equivalent stiffness and excitation amplitude of the system, these related parameters are more difficult to test, especially the equivalent damping coefficient and equivalent stiffness, etc., so in the practical engineering application, the vibration energy of the system is generally to characterize with the vibration amplitude (displacement, velocity, acceleration). The lower the amplitude is, the smaller the vibration energy and the larger the damping energy consumption. In order to simplify the characterization of the vibration suppression effect, it is more convenient to describe the vibration suppression effect by the relative value of amplitude, i.e. the dimensionless VRR, with the cavity system as the reference object.

### Particle damping parameter screening method

In order to explore the internal relationship between TEC and VRR, this paper only discusses some related parameters of a single particle damper, but does not involve that of multiple particle dampers, particle materials, system noise, rotating excitation and other parameters, as well as the complex working conditions of multi-damper, multi-factor and multi-level. The simulation and test of particle damping require a lot of work, even if huge manpower and material resources are spent, it is difficult to complete all research projects in a short period of time. Therefore, appropriate analysis methods should be selected in the follow-up study to reduce the number of simulation and test as much as possible, so that a small amount of simulation and test data can reflect the comprehensive research results and be representative, and a high confidence conclusion can be obtained.

In a sense, simulation is also an experiment, but the experiment equipment is a computer. The screening of particle damping parameters can adopt orthogonal simulation method to expand the scope of the parameter selection, make each representative and comprehensive comparison between the simulation and test groups, solve theoretically the test times and the actual number of contradiction to a certain extent, and overcome the contradiction between the number of tests needed in theory and the number of actual tests, as well as the contradiction between the number of actual tests and the requirement to master the internal laws of things. On the basis of orthogonal simulation results, the bench test can be carried out to obtain the interaction results of various factors, which can greatly shorten the research time and save the cost, and create favorable conditions for further research.

## Conclusion

Research results on longitudinal vibration suppression of underwater vehicle shafting based on particle damping are shown as follows:

Firstly, simulation and test results show that under the rotating speed and longitudinal excitation conditions, the energy consumption caused by the collision and friction between particles and damper walls and between particles can inhibit the longitudinal vibration of the system, and the suppression levels are constrained by the parameters such as mass filling ratio, cavity length, rotating speed, particle radius, excitation frequency and amplitude, especially, the first four parameters have greater influence. Vibration suppression effect is the best at the main frequency 5 Hz.

Secondly, the influence effects of mass filling ratio, cavity length, rotating speed, particle radius, excitation frequency and amplitude on the system vibration suppression indicate that variation trend of both TEC and VRR are almost the same at *n* = 90 r/min, and the energy consumption simulation model of particle damping based on DEM is reasonable and simulation data reliable.

Thirdly, the intrinsic relationship between energy consumption of particle damping and vibration energy was revealed and the method of evaluating longitudinal vibration suppression effect based on TEC and VRR presented.

## Data Availability

The data used to support the findings of this study are included in the article.
